# Older People With Dementia Running a Pop-Up Restaurant: How to Use Reality Shows to Reduce the Stigma of Dementia

**DOI:** 10.1093/geroni/igab046.685

**Published:** 2021-12-17

**Authors:** Wenqian Xu

**Affiliations:** Linköping University, Norrköping, Ostergotlands Lan, Sweden

## Abstract

The present study focused on a Chinese reality show, Forget Me Not Café, which brought together five older people (aged 65 and older) living with dementia to run a pop-up restaurant and intended to reduce the stigma of dementia. The study aims to explore how the reality-show participants describe dementia in older people and how their views relate to the macrosocial context of dementia and older people. This study performed a thematic discourse analysis on the written and spoken content about dementia in older people (or later life) presented in the reality show. Four discursive themes were identified including: (1) age is a risk factor for dementia; (2) early signs and symptoms of dementia in older people deserve attention; (3) putting pressure on family caregivers of people with dementia; (4) expectations to maintain social engagement and slow down the development of dementia. This study also found that the views of the reality-show participants highlight the capability of older people with dementia to communicate effectively and live with the condition, their personal goals of sustaining a happy, meaningful and sociable life, as well as their actions to positively influence personal circumstances. The results of this study indicate that this reality show might help reduce the stigma of dementia and empower older people living with dementia, while it also tends to stress the responsibility for care on family carers and shift the responsibility of managing the dementia-related challenges to older people living with dementia.

